# Nationwide survey of medical student interest in and exposure to aerospace medicine

**DOI:** 10.1038/s41526-023-00287-y

**Published:** 2023-06-14

**Authors:** Semran B. Thamer, Joseph Bello, Mirjana Stevanovic, Dennis Obat, Jay C. Buckey

**Affiliations:** 1 Geisel School of Medicine at Dartmouth, 1 Rope Ferry Rd, Hanover, NH 03755 USA; 2 Space Medicine Innovations Laboratory, Geisel School of Medicine at Dartmouth, One Medical Center Drive, Lebanon, NH 03756 USA

**Keywords:** Occupational health, Education

## Abstract

Aerospace Medicine is experiencing a renaissance. Commercial spaceflight is now a reality, meaning that individuals with a variety of medical conditions will be flying in space. NASA has Mars plans, and SpaceX plans to send humans to Mars within the next decade, so today’s medical students may be future physicians on these crews. Considering these developments, we evaluated interest in and exposure to Aerospace Medicine among medical students in the United States (US). A 19-question anonymous multiple-choice questionnaire was emailed to current medical students throughout the US. Information about demographics, career and research interests in aerospace medicine, opportunities available at students’ respective institutions, and possible avenues for supporting students’ interests was collected and analyzed. One thousand two hundred forty-four students (490 men, 751 women, 3 other) with a mean age of 25.8 ± 3.0 years from 60 institutions completed the questionnaire. Most respondents expressed an interest in learning about aerospace medicine during their training. A strong interest in research, as well as career opportunities, exists despite the majority of students reporting minimal access to opportunities to get involved in the field at most of the surveyed institutions. With growing interest and an expected increase in demand for physicians with a background in aerospace medicine, medical schools may be able to support students by increasing access to opportunities.

## Introduction

Aerospace Medicine is a specialty within preventative and occupational medicine focusing on the health of pilots, astronauts, flight crewmembers, and passengers involved in air and space travel^1^. The development of aerospace medicine as an officially recognized specialty closely followed the development of reliable airplanes in the early 20^th^ century^2^. World War One was the first major conflict that involved the large-scale use of aerial warfare and led to the rapid development of reliable aircraft^3^. During that time, as technology in aviation grew, physicians began to develop an understanding of the physiology of flight. Commercial aviation became increasingly feasible, and the importance of medicine and public health to support this growing need became increasingly recognized. As a result, the American Board of Preventive Medicine and Public Health developed a pathway whereby physicians could become trained in aerospace medicine^4^.

The rapid development of aerospace medicine as a field was shortly followed by growing interest in space travel^5^. When the National Aeronautics and Space Administration (NASA) began to solidify plans for manned space flight, Aviation Medicine evolved to become what is now known as Aerospace Medicine^2^. This transition led to the growth and development of a wide array of career opportunities in the field, with research and clinical experiences in both military and civilian settings. Since then, there have been significant advances in space technology, and commercial spaceflight is now a reality^6^. Civilians representing a wide range of ages and backgrounds have already begun to travel to space, and the demand for commercial spaceflight is projected to increase exponentially^7,8^. As a result, more aerospace medicine physicians are needed to support this growing demand.

As spaceflight enters a new era of sustainable human exploration, the field of aerospace medicine will need to follow. The success of future spaceflight will depend on training physicians to address the unique challenges and physiological adaptations in space^9^. To date, there are five accredited aerospace medicine programs in the United States—three military programs (the Air Force Program at Wright-Patterson, the Naval Aerospace Medical Institute, and the Army program at Fort Rucker) and two civilian programs (Mayo Clinic College of Medicine and University of Texas Medical Branch)^10^. With organizations like NASA, SpaceX, and Blue Origin planning numerous missions to send humans to space within the next decade, today’s medical students will likely be the future physicians involved with these crews^11–13^.

On average, only three to four civilian aerospace medicine physicians graduate each year in the United States^14^. While this number will likely increase as demand grows, the level of interest among today’s medical students in pursuing a career in the field has not been studied. Previous research shows most medical students are amenable to career suggestions, and so early exposure may increase interest in the growing field^15^. However, it is not clear if exposure to aerospace medicine is offered at most medical schools. In this research, we evaluated interest in aerospace medicine among medical students and gauged opportunities for exposure to the field in medical schools throughout the United States.

## Results

### Responses to questionnaire

A total of 1244 respondents completed the questionnaire — 490 men, 751 women, and 3 ‘other’ with a mean age of 25.8 ± 3.0 years. Of the 155 institutions contacted, respondents represented 60 institutions across the United States (38.7% response rate). 391 (31.4%) respondents were first-years, 318 (25.6%) second-years, 265 (21.3%) third-years, and 240 (19.3%) fourth-years. A minority (30, 2.4%) selected ‘other’ as their year in medical school. 1,036 (83.3%) respondents were MD students, and 208 (16.7%) were DO students. 3 (0.2%) identified as American Indian or Alaska Native, 245 (19.7%) Asian, 55 (4.4%) Black or African American, 4 (0.3%) Native Hawaiian or other Pacific Islander, 841 (67.6%) White, and 91 (7.3%) ‘other’ (Table 1).

**Table 1:** Respondent demographics.

	Overall (*N*^a^ = 1244)
*Age (years)*	
Mean ± SD^b^	25.8 ± 3.0
Median	25.0
*Sex*	
Male	490 (39.4%)
Female	751 (60.4%)
Other	3 (0.2%)
*Race*	
American Indian or Alaska Native	3 (0.2%)
Asian	245 (19.7%)
Black or African American	55 (4.4%)
Native Hawaiian or Other Pacific Islander	4 (0.3%)
White	841 (67.6%)
Other	91 (7.3%)
*Year in school*	
M1/First year	391 (31.4%)
M2/Second year	318 (25.6%)
M3/Third year	265 (21.3%)
M4/Fourth year	240 (19.3%)
Other	30 (2.4%)

^a^Sample size.

^b^Standard deviation.

Six hundred and twelve (49.2%) reported having never heard of aerospace medicine as a field, and 944 (75.9%) reported that they did not know that it is an official medical specialty. Men were significantly more likely to report knowledge of aerospace medicine as a field and specialty (*p* < 0.001). First-years had the highest proportion of students reporting no knowledge of aerospace medicine, while second, third, and fourth years reported significantly more knowledge about the field (*p* < 0.05). Totally, 1008 (81.0%) of respondents rated the importance of physician involvement in space exploration as moderately to extremely important, and there was no significant difference across years of school or sex (Table 2). There was no statistically significant association between knowledge of aerospace medicine as a field or medical specialty and respondents’ rating of the importance of physician involvement in space exploration (*p* = 0.168).

**Table 2:** Summary of responses.

Survey question	Number	% of total
*Have you ever heard of Aerospace Medicine as a field?*		
Yes	632	50.8%
No	612	49.2%
*Did you know that Aerospace Medicine is an official medical specialty?*		
Yes	277	22.3%
No	944	75.9%
*If you had the opportunity, would you be interested in learning more about Aerospace Medicine?*		
Yes	1061	85.3%
No	182	14.6%
*If given the opportunity, what aspects of Aerospace Medicine would you be interested in pursuing?*		
Research	208	16.7%
Caring for the health of astronauts	455	36.6%
Becoming an astronaut physician	484	38.9%
*Does your institution have an Aerospace Medicine group? (i.e., interest group)*		
Yes	65	5.2%
No/unsure	1175	94.4%
*Does your institution offer an Aerospace Medicine elective or enrichment opportunity?*		
Yes	33	2.7%
No/unsure	1201	96.5%
*At your institution, do you have access to opportunities to get involved with conducting or participating in Aerospace Medicine research?*		
Yes	55	4.4%
No/unsure	1167	93.9%
*How important do you think it is for physicians to be involved in space exploration?*		
Not at all important	31	2.5%
Slightly important	181	14.5%
Moderately important	289	23.2%
Very important	429	34.5%
Extremely important	290	23.3%

One thousand sixty-one (85.3%) respondents expressed interest in learning more about aerospace medicine if given the opportunity, and this was not significantly associated with prior knowledge of aerospace medicine as a field or medical specialty (*p* = 0.303). Among those who expressed interest in an aerospace medicine career, 208 (16.7%) specified an interest in research, 455 (36.5%) in caring for the health of astronauts, and 484 (38.9%) in becoming an astronaut physician (Table 2). Men were more likely to report interest in learning more about aerospace medicine (*p* < 0.001).

Sixty-five (5.2%) respondents indicated that they had access to a Space Medicine interest group, 33 (2.7%) to an elective/enrichment opportunity, and 55 (4.4%) to research opportunities at their respective institutions (Table 2). The 153 respondents who reported having access to an interest group, elective/enrichment opportunity, or research opportunities represented 8 distinct institutions. Other respondents from the same 8 institutions did not report having access to these opportunities. Totally, 43.1% of those who had access to an interest group reported that it helped them establish or solidify their interest in the field. Among those who did not have access to an interest group, elective/enrichment, or research opportunities, 484 (79.1%) reported that they would have joined or participated in these activities if offered at their institutions.

Respondents who had access to an interest group, elective/enrichment, or research opportunities were more likely to report interest in an aerospace medicine career than those who did not (*p* < 0.0001). Of the 159 (12.8%) respondents who reported that they were probably or definitely considering an aerospace medicine career, 20 (12.6%) had access to an interest group, 12 (7.5%) to an elective/enrichment, and 15 (9.4%) to research opportunities.

## Discussion

To the best of our knowledge, this is the first study evaluating interest in and exposure to aerospace medicine among medical students in the United States. The sex, age, and race distribution of the respondents was diverse. In this cohort of medical students from across the United States, we found that significant interest in the field exists, while exposure to the field during medical school is rare. This apparent gap between interest and opportunities for exposure warrants important consideration as we approach a new era in spaceflight and aerospace medicine.

According to a report published by the Association of American Medical Colleges, the United States is projected to experience a shortage of between 37,800 and 124,000 physicians by 2034^16^. The shortage is expected to disproportionately affect primary care—one of the most common pathways toward specialty training in aerospace medicine. A potential shortage of aerospace medicine physicians may impede already planned missions to send humans to space in the next few decades. Today’s medical students are uniquely positioned to graduate and complete their training by the time NASA plans to send humans to Mars in 2030 or when other organizations send missions to the Moon and low Earth orbit in the next decade^11–13^.

The findings of the study reassuringly suggest that significant interest in the field exists among medical students, and, as a result, lack of interest is unlikely to impede meeting the projected increase in demand for aerospace medicine physicians. The choice of physician specialty, however, is a complex decision that includes many influencing factors^17^. Research shows that as many as 70% of students change their mind about specialty choice during medical school, with early exposure and the presence of opportunities in the field identified as key factors in influencing these decisions^18^.

In this study, the majority of respondents did not report having exposure or access to learning and research opportunities in aerospace medicine during medical school. Those who did report significantly more interest in pursuing a career in the field. This suggests that increased exposure or access to opportunities may, in turn, increase interest. Thus, addressing the gap between interest in, and exposure to aerospace medicine, may help address the growing need for physicians trained in the field. The results of this study also showed that students from the same institution differed in their responses about awareness of opportunities available to them. This suggests that awareness about opportunities within an institution may be limited to a subset of students. Additionally, males were more likely to report knowledge of aerospace medicine as well as interest in learning more about the field. Interventions to increase awareness among women and ‘other’ sexes may promote greater diversity in aerospace medicine.

This study has limitations. Its voluntary, survey-based design means there is sampling and response bias, so the responses may not be representative of all medical students in the United States. Nevertheless, the study captured the responses of a large, geographically diverse cohort of medical students from a range of demographic backgrounds reflective of the current medical student body. Additionally, students who were willing to take the survey may have different attitudes about aerospace medicine than those who were not willing to do so. The cross-sectional nature of this study’s design precludes any causal inferences to be made about the relationship between medical student interest and exposure to opportunities in the field. Additionally, the questionnaire may have had an embedded bias towards space operations, while the field of aerospace medicine encompasses a broad scope of operations that may not necessarily involve space. Further studies exploring a wider breadth of operations aerospace medicine encompasses may provide more information about student interest in the field.

This study demonstrates that while significant interest in aerospace medicine exists among medical students in the United States, there is an apparent gap between interest and exposure to opportunities in the field in medical schools across the nation. Medical schools may consider increasing awareness of unique career opportunities like aerospace medicine among medical students through career exploration activities facilitated by the institutions. Addressing this gap may help facilitate increased interest in the field and decrease the risk of aerospace medicine physician shortages in the face of a projected increase in demand. Given that a limited number of board-certified aerospace medicine trainees are admitted each year, increasing capacity at existing programs or establishing novel pathways for career opportunities in the field may also be necessary to facilitate the future expansion of physicians involved in aerospace medicine. Further research is needed to validate the findings of this study and assess the efficacy of early exposure to opportunities in the field in promoting medical student interest.

## Methods

### Questionnaire design and distribution

This questionnaire study was reviewed and approved by the Dartmouth College Committee for the Protection of Human Subjects, and informed consent was obtained from each participant. We used Qualtrics to create a 19-item web-based questionnaire tool to collect demographic information, determine the level of interest in aerospace medicine, and assess opportunities for exposure to the field. Participation in the questionnaire was voluntary, and all responses were kept anonymous and confidential. No identifying information was collected.

The questionnaire was disseminated via email to medical students across the US from October 1st, 2021 to December 1st, 2021. A list of all 155 American Association of Medical Colleges (AAMC) in the United States was generated, and for each school on the list, the Student Affairs offices were contacted via email. They were given a description of the study and asked to distribute the questionnaire to their respective institution’s entire medical student body. The decision to distribute the questionnaire was made on the administrative level by the institutions’ Student Affairs offices. Institutions that were not represented by this study’s questionnaire either did not respond to a request to distribute the questionnaire or stated that it was against their institutional policies to distribute external questionnaires. The questionnaire was self-administered and accessed by participants via an electronic link. Only one submission per respondent was allowed by the questionnaire’s design.

Responses were collected through the questionnaire’s embedded secure online portal, and summary descriptive statistics were compiled using Qualtrics. We used logistic regression for hypothesis testing. All statistical tests were two-sided, and statistical significance was considered at *p* < 0.05. All statistical tests, as well as data cleaning and pre-processing, were implemented using R. Incomplete responses were excluded from the analysis. We identified respondents who were missing a value for the year of medical education, sex, age, and/or race—these respondents were also missing all answers to the remainder of the questionnaire and were therefore removed from the analysis.

### Reporting summary

Further information on research design is available in the Nature Research Reporting Summary linked to this article.

## Supplementary information

Reporting Summary

## Data Availability

The data that support the findings of this study are available from the corresponding author, [S.T.], upon reasonable request.
