# Supplementary material for: Nationwide survey of medical student interest in and exposure to aerospace medicine
Source: NPJ Microgravity. 2023 Jun 14;9:44. doi: 10.1038/s41526-023-00287-y (PMC10267131; doi:10.1038/s41526-023-00287-y)
Supplement: Supplementary file 1 — Reporting Summary [file 41526_2023_287_MOESM1_ESM.pdf]

nature portfolio

Corresponding author(s): NPJMGRAV-00895R

Last updated by author(s): 4/2/2023

Reporting Summary

Nature Portfolio wishes to improve the reproducibility of the work that we publish. This form provides structure for consistency and transparency in reporting. For further information on Nature Portfolio policies, see our Editorial Policies and the Editorial Policy Checklist.

Statistics

For all statistical analyses, confirm that the following items are present in the figure legend, table legend, main text, or Methods section.

n/a	Confirmed	
☐	☒	The exact sample size (*n*) for each experimental group/condition, given as a discrete number and unit of measurement
☐	☒	A statement on whether measurements were taken from distinct samples or whether the same sample was measured repeatedly
☐	☒	The statistical test(s) used AND whether they are one- or two-sided<br>*Only common tests should be described solely by name; describe more complex techniques in the Methods section.*
☒	☐	A description of all covariates tested
☒	☐	A description of any assumptions or corrections, such as tests of normality and adjustment for multiple comparisons
☐	☒	A full description of the statistical parameters including central tendency (e.g. means) or other basic estimates (e.g. regression coefficient) AND variation (e.g. standard deviation) or associated estimates of uncertainty (e.g. confidence intervals)
☐	☒	For null hypothesis testing, the test statistic (e.g. *F*, *t*, *r*) with confidence intervals, effect sizes, degrees of freedom and *P* value noted<br>*Give P values as exact values whenever suitable.*
☒	☐	For Bayesian analysis, information on the choice of priors and Markov chain Monte Carlo settings
☒	☐	For hierarchical and complex designs, identification of the appropriate level for tests and full reporting of outcomes
☒	☐	Estimates of effect sizes (e.g. Cohen's *d*, Pearson's *r*), indicating how they were calculated

Our web collection on statistics for biologists contains articles on many of the points above.

Software and code

Policy information about availability of computer code

Data collection	R 4.2.1 was used to conduct statistical analysis
Data analysis	*Provide a description of all commercial, open source and custom code used to analyse the data in this study, specifying the version used OR state that no software was used.*

For manuscripts utilizing custom algorithms or software that are central to the research but not yet described in published literature, software must be made available to editors and reviewers. We strongly encourage code deposition in a community repository (e.g. GitHub). See the Nature Portfolio guidelines for submitting code & software for further information.

Data

Policy information about availability of data

All manuscripts must include a data availability statement. This statement should provide the following information, where applicable:

- Accession codes, unique identifiers, or web links for publicly available datasets
- A description of any restrictions on data availability
- For clinical datasets or third party data, please ensure that the statement adheres to our policy

The data that support the findings of this study is available from the corresponding author, [ST], upon reasonable request.

Human research participants

Policy information about studies involving human research participants and Sex and Gender in Research.

Reporting on sex and gender	Sex was self-reported by participants of our survey. Gender was not collected.
Population characteristics	1,244 students (490 men, 751 women, 3 other) with a mean age of 25.8 ± 3.0 years from 60 institutions completed the questionnaire.
Recruitment	The questionnaire was disseminated via email to medical students across the US from October 1st 2021 to December 1st 2021. A list of all 155 American Association of Medical Colleges (AAMC) in the United States was generated, and for each school on the list, the Student Affairs offices were contacted via email. They were given a description of the study and asked to distribute the questionnaire to their respective institution's entire medical student body. The decision to distribute the questionnaire was made on the administrative level by the institutions' Student Affairs offices to minimize self selection bias. Selection bias is still possible given that participation was voluntary. Nonetheless, this study included a large number of participants across the nation.
Ethics oversight	Dartmouth College Committee for the Protection of Human Subjects

Note that full information on the approval of the study protocol must also be provided in the manuscript.

Field-specific reporting

Please select the one below that is the best fit for your research. If you are not sure, read the appropriate sections before making your selection.

☐ Life sciences ☒ Behavioural & social sciences ☐ Ecological, evolutionary & environmental sciences

For a reference copy of the document with all sections, see nature.com/documents/nr-reporting-summary-flat.pdf

Behavioural & social sciences study design

All studies must disclose on these points even when the disclosure is negative.

Study description	Qualitative cross-sectional survey study
Research sample	Medical Students in the United States
Sampling strategy	See above regarding recruting
Data collection	The questionnaire was self-administered and accessed by participants via an electronic link. Only one submission per respondent was allowed by the questionnaire's design. Responses were collected through the questionnaire's embedded secure online portal, and summary descriptive statistics were compiled using Qualtrics.
Timing	The questionnaire was disseminated via email to medical students across the US from October 1st 2021 to December 1st 2021.
Data exclusions	We identified respondents who were missing a value for year of medical education, sex, age, and/or race —these respondents were also missing all answers to the remainder of the questionnaire, and were therefore removed from the analysis. 79 responses were removed from the study.
Non-participation	Of the 155 institutions that were contacted, respondents represented 61 institutions across the United States (39.4% response rate).
Randomization	This was not a randomized study, rather survey based

Reporting for specific materials, systems and methods

We require information from authors about some types of materials, experimental systems and methods used in many studies. Here, indicate whether each material, system or method listed is relevant to your study. If you are not sure if a list item applies to your research, read the appropriate section before selecting a response.

Materials & experimental systems

n/a	Involved in the study
☒	☐ Antibodies
☒	☐ Eukaryotic cell lines
☒	☐ Palaeontology and archaeology
☒	☐ Animals and other organisms
☒	☐ Clinical data
☒	☐ Dual use research of concern

Methods

n/a	Involved in the study
☒	☐ ChIP-seq
☒	☐ Flow cytometry
☒	☐ MRI-based neuroimaging
